# Supplementary material for: Causal Relationship between Aging and Anorexia Nervosa: A White-Matter-Microstructure-Mediated Mendelian Randomization Analysis
Source: Biomedicines. 2024 Aug 16;12(8):1874. doi: 10.3390/biomedicines12081874 (PMC11351342; doi:10.3390/biomedicines12081874)
Supplement: Supplementary file 1 [file biomedicines-12-01874-s001.zip › Supplementary Figure/Figure S4/Singe SNP plot of MR analysis for TL to ubm-b-1907.pdf]

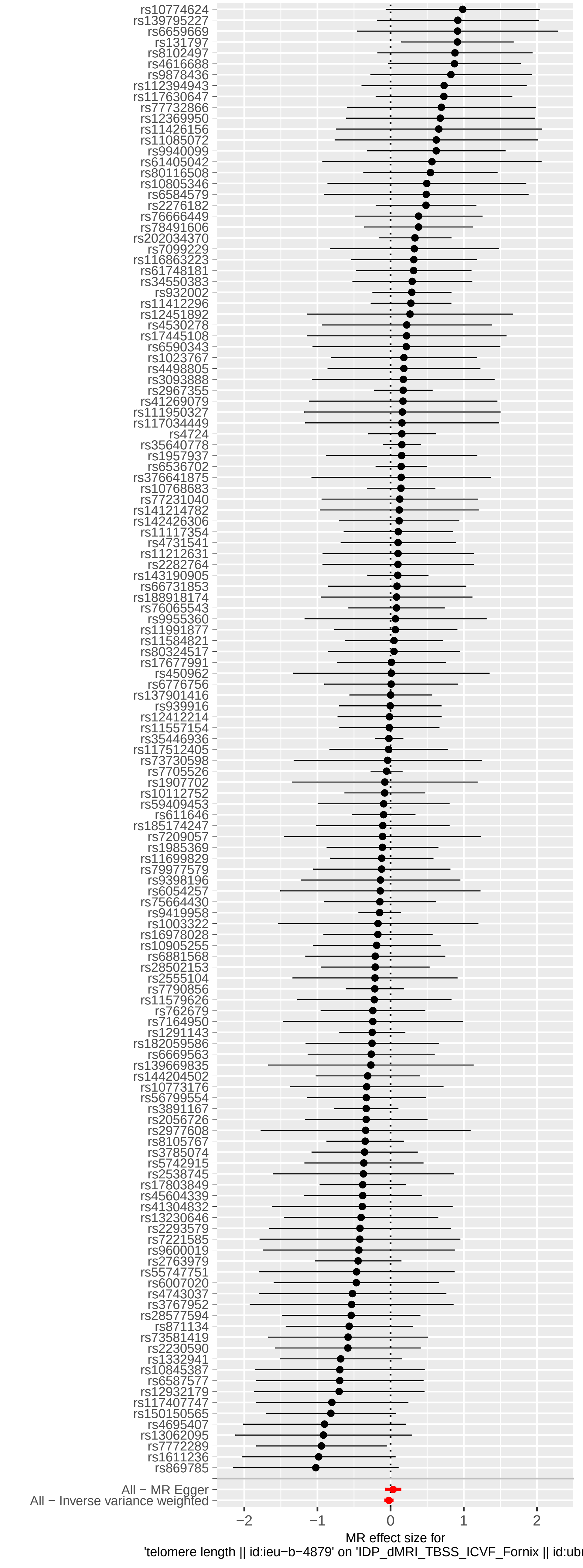

rs10774624
rs139795227
rs6659669
rs131797
rs8102497
rs4616688
rs9878436
rs112394943
rs117630647
rs77732866
rs12369950
rs11426156
rs11085072
rs9940099
rs61405042
rs80116508
rs10805346
rs6584579
rs2276182
rs76666449
rs78491606
rs202034370
rs7099229
rs116863223
rs61748181
rs34550383
rs932002
rs11412296
rs12451892
rs4530278
rs17445108
rs6590343
rs1023767
rs4498805
rs3093888
rs2967355
rs41269079
rs111950327
rs117034449
rs4724
rs35640778
rs1957937
rs6536702
rs376641875
rs10768683
rs77231040
rs141214782
rs142426306
rs11117354
rs4731541
rs11212631
rs2282764
rs143190905
rs66731853
rs188918174
rs76065543
rs9955360
rs11991877
rs11584821
rs80324517
rs17677991
rs450962
rs6776756
rs137901416
rs939916
rs12412214
rs11557154
rs35446936
rs117512405
rs73730598
rs7705526
rs1907702
rs10112752
rs59409453
rs611646
rs185174247
rs7209057
rs1985369
rs11699829
rs79977579
rs9398196
rs6054257
rs75664430
rs9419958
rs1003322
rs16978028
rs10905255
rs6881568
rs28502153
rs2555104
rs7790856
rs11579626
rs762679
rs7164950
rs1291143
rs182059586
rs6669563
rs139669835
rs144204502
rs10773176
rs56799554
rs3891167
rs2056726
rs2977608
rs8105767
rs3785074
rs5742915
rs2538745
rs17803849
rs45604339
rs41304832
rs13230646
rs2293579
rs7221585
rs9600019
rs2763979
rs55747751
rs6007020
rs4743037
rs3767952
rs28577594
rs871134
rs73581419
rs2230590
rs1332941
rs10845387
rs6587577
rs12932179
rs117407747
rs150150565
rs4695407
rs13062095
rs7772289
rs1611236
rs869785
All − MR Egger
All − Inverse variance weighted
−2
−1
0
1
2
MR effect size for
'telomere length || id:ieu−b−4879' on 'IDP_dMRI_TBSS_ICVF_Fornix || id:ubr
